# Supplementary material for: Anti-Cancer and Anti-Inflammatory Activities of a Short Molecule, PS14 Derived from the Virulent Cellulose Binding Domain of Aphanomyces invadans, on Human Laryngeal Epithelial Cells and an In Vivo Zebrafish Embryo Model
Source: Molecules. 2022 Oct 28;27(21):7333. doi: 10.3390/molecules27217333 (PMC9654460; doi:10.3390/molecules27217333)
Supplement: Supplementary file 1 [file molecules-27-07333-s001.zip › molecules-1966650-supplementary.pdf]

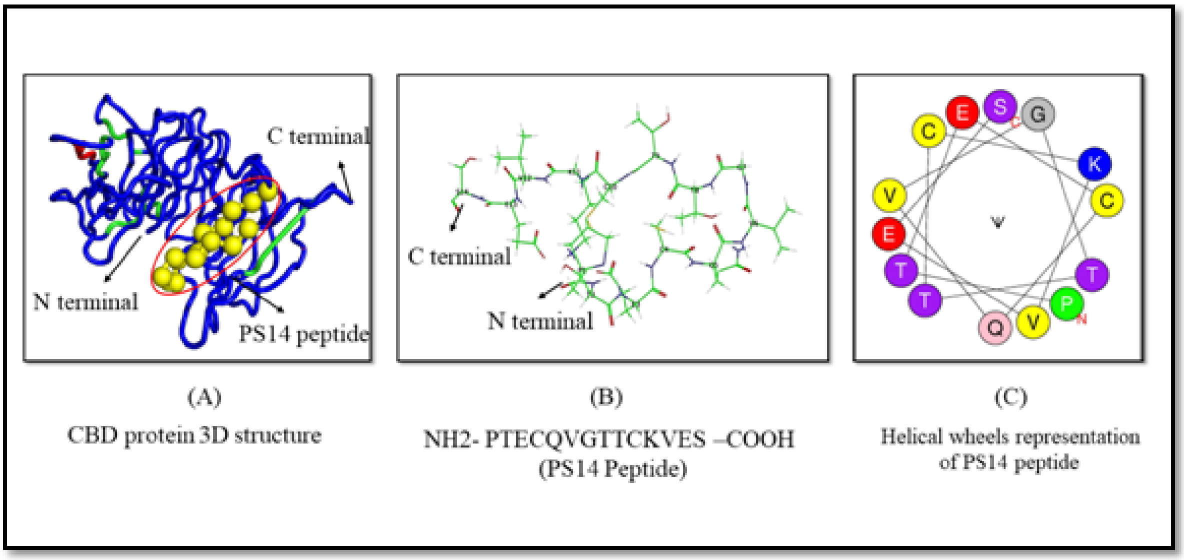

E-Suppl. Fig. S1. Bioinformatic analysis of CBD protein and its derived molecule, PS14.

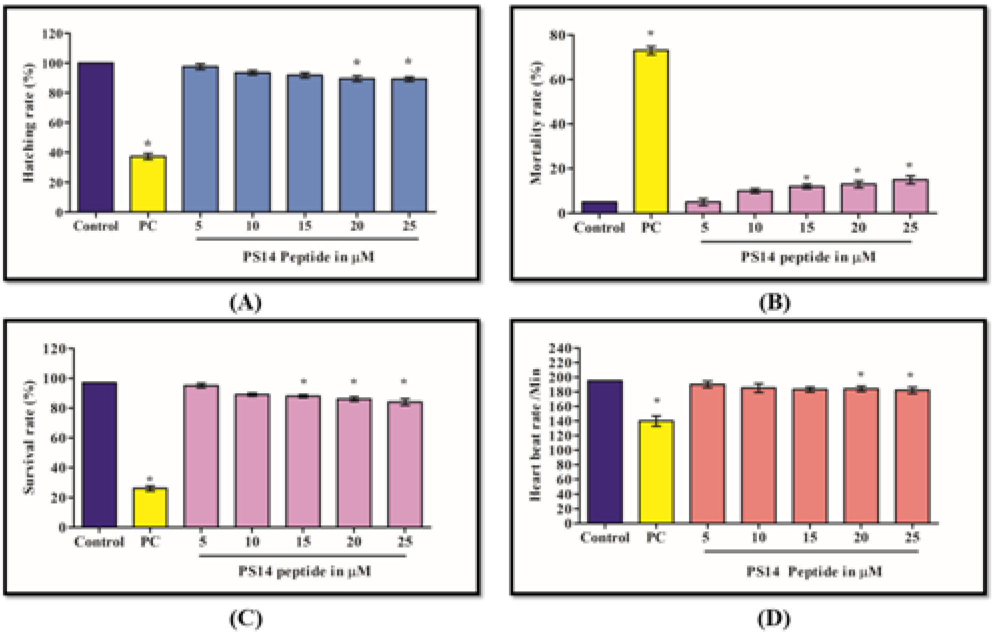

E-Suppl. Fig. S2. *In-vivo* toxicity of zebrafish larvae treated with five different concentrations of PS14. The untreated individuals were considered as control and larvae treated with 1 mM of H_2O_2 was condisered as positive control (PC). **(A)** The hatching rate was calculated at 48 hpf; **(B)** The mortality rate was calculated at 96 hpf; **(C)** The survival rate was calculated at 96 hpf; and **(D)** The heart rate was calculated at 72 hpf. The data was compared with the untreated control, which showed a level of significance at $p < 0.05$ that was represented by *****. The data was provided as mean ± SD of three independent experiments.

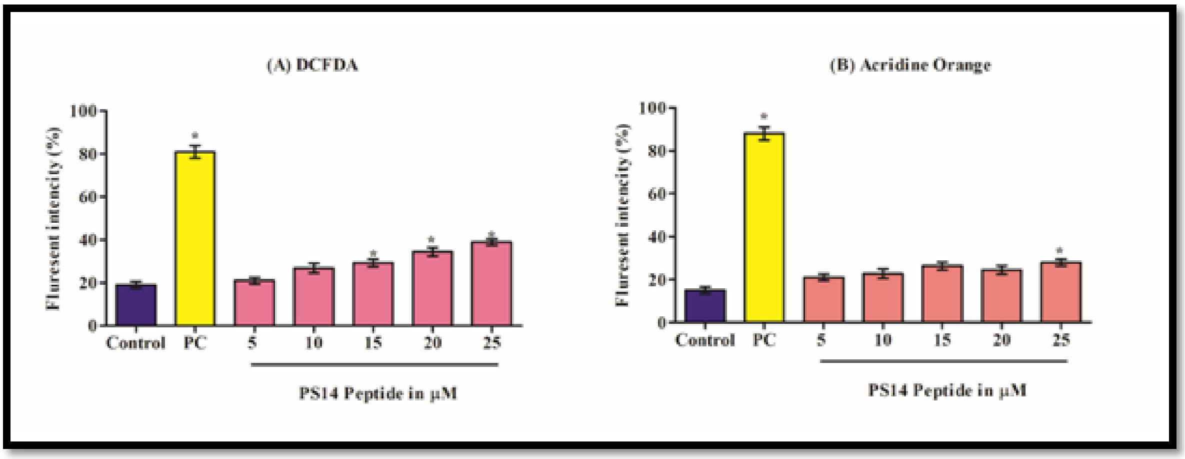

E-Suppl. Fig. S3. Quantitative measurement of the fluorescent intensity in zebrafish larvae, images processed in image J software. **(A)** DCFDA staining and **(B)** Acridine orange stating assay. Experiments were performed in triplicates and the data were expressed as mean ± SD of three replicates. The asterix (*****) represents the statistical significance at $p < 0.05$ compared to the control.

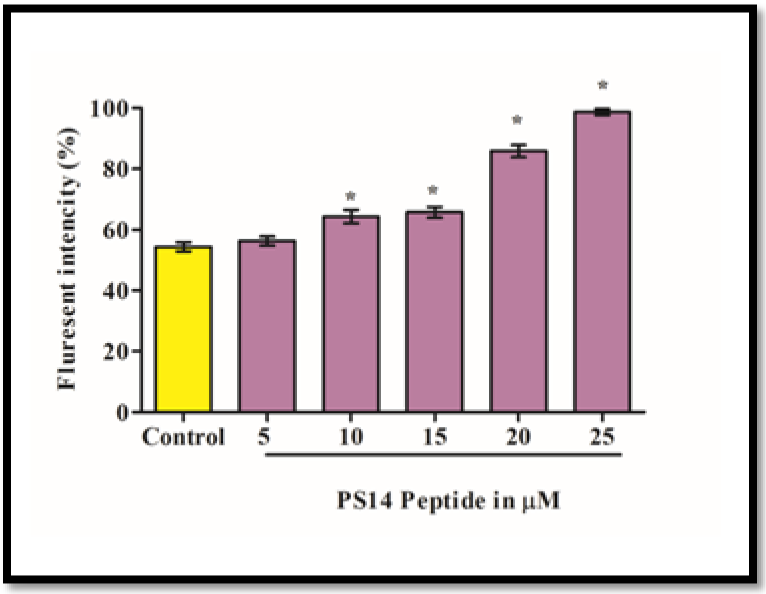

E-Suppl. Fig. S4. *In-vitro* ROS generation potential of PS14 against Hep-2 cells. The bar showed the quantitative measurement of the fluorescent intensity; images are processed in image J software. Experiments were performed in triplicate and the data were expressed as mean ± SD. The asterix (*****) represents the statistical significance at $p < 0.05$ compared to the control.
